# Faces in a Crowd: High Socially Anxious Individuals Estimate that More People Are Looking at Them than Low Socially Anxious Individuals

**DOI:** 10.1371/journal.pone.0106400

**Published:** 2014-09-10

**Authors:** Olivia C. Bolt, Anke Ehlers, David M. Clark

**Affiliations:** 1 Department of Psychology, King's College London and National Institute for Health Research (NIHR) Mental Health Biomedical Research Centre at South London and Maudsley NHS Foundation Trust, London, United Kingdom; 2 Department of Experimental Psychology, University of Oxford, Oxford, Oxfordshire, United Kingdom; Harvard Medical School, United States of America

## Abstract

**Background:**

People with social anxiety disorder are afraid of being scrutinized by others and often feel that they are the excessive focus of other people's attention. This study investigated whether, when compared to low socially anxious individuals, high socially anxious individuals overestimate the proportion of people in a crowd who are observing them. It was hypothesized that any potential overestimation would be modulated by self-focused attention.

**Method:**

Forty-eight high and 48 low socially anxious participants performed a “faces in a crowd” computer task during which they briefly saw matrices of faces, which varied in terms of the proportion of people who were looking at them. Participants estimated the proportion of people who were looking at them. The task was performed once with mirrors present (to induce an enhanced self-focused state) and once without mirrors present (neutral state).

**Results:**

Participants' subjective estimates and the objective proportion of faces looking towards them were strongly correlated in both the high and low socially anxious groups. However, high socially anxious participants estimated that more people were looking at them than low socially anxious participants. In the first phase of the experiment, but not in the later phases, this effect was magnified in the mirror condition.

**Discussion:**

This study provides preliminary evidence of a social anxiety related perceptual difference that may be amplified by self-focused attention. Clinical implications are discussed.

## Introduction

Social anxiety disorder (SAD) is characterized by an extreme fear of being negatively evaluated by other people [1]. *Fear of being observed* is a common feature of SAD [2]. While in social situations, individuals with SAD have the impression that they are the excessive focus of other people's attention. There are two possible explanations for this impression. First, the impression may reflect reality. Individuals with SAD may attract other people's attention to a greater extent, perhaps because of certain aspects of their behavior (such as staying on the edge of groups, or being socially withdrawn), or because some of the symptoms of their anxiety are visible (e.g. sweating, or blushing). Some studies, e.g. [3], have found that individuals with SAD are rated as performing noticeably differently in social situations, but this effect has not always been replicated [4], and it is also not known whether such differences in performance would attract other people's attention. Second, individuals with SAD may differ from individuals without SAD in their perception of the extent to which they are the focus of other people's attention. In particular, they may be prone to perceive a greater proportion of people looking at them than individuals without SAD even when there is no objective difference. The present study examined the second possibility.

Recent research into the perception of another person's gaze has provided some support for the view that individuals with SAD are more likely to think another individual is looking at them than non-clinical controls (for a review, see [5]). In the “cone of gaze” paradigm individuals with SAD and non-clinical controls were asked to rotate the eyes of a virtual head that were initially looking at them to the point when they felt the eyes were about to stop looking at them. People with SAD showed a wider cone of gaze than non-clinical controls [6], [7]. This difference was also present when a real actor was used instead of a virtual head. After a course of cognitive behavioral therapy (CBT), the difference in cone of gaze between individuals with SAD and non-clinical controls was no longer statistically significant [7]. Although the cone of gaze paradigm shows that under some circumstances people with SAD are more likely to think they are being looked at by another person, its ecological validity is somewhat restricted. It models a single individual watching you out of the corner of his/her eyes. Clinically, individuals with SAD rarely mention being concerned that this is happening. Instead, they seem more concerned that people are staring directly at them and are particularly troubled by the feeling that a whole crowd of people may be looking at them. So far, no study has investigated what underlies the common report of patients with SAD that “everybody is staring at me”, for example when they are entering a room full of people, or when they are walking down a crowded street.

The present study explored this phenomenon by creating multiple faces visual displays that were presented briefly and varied in terms of the number of people who were looking at participants. High and low socially anxious participants were asked to estimate the proportion of people who were looking at them. With this multiple faces in a crowd paradigm, we tried to capture the first impression process that a person is going through when entering a new social situation. Such first impressions are very important for people with social anxiety as they often determine whether the person looks away, escapes, or otherwise disengages from the social situation.

Cognitive models of SAD [8]–[10] propose that enhanced self-focused attention and monitoring in social situations is one of the key maintenance factors for SAD. One might deduce from this theoretical position the hypothesis that if people with high levels of social anxiety estimate that more people are looking at them, this may be because they are mistaking self-observation for observation by others. The present study investigated this possibility by the use of a mirror manipulation. Previous research has shown that placing a mirror in the experimental room increases self-focused attention and self-consciousness [11].

## Method

### Overview

Participants performed a “faces in a crowd” task on a computer. Matrices of faces were briefly presented with some faces looking towards the participant and others looking sideways or down. The participants' task was to estimate the proportion of faces that were looking towards them. The task was performed twice, once with mirrors present and once without the mirrors [11]. Two parallel versions of the task were used. Order of mirror present/absent and task version were counter-balanced within high and low socially anxious participants. The study was approved by the King's College Research Ethics Committee (Reference number: *PNM/08/09-124)*.

### Participants

Forty-eight high socially anxious and 48 low socially anxious participants completed the experiment. Participants were invited to take part if they scored in the top or bottom 25% of a student distribution of the 12-item version of the Brief Fear of Negative Evaluation Scale (bFNES; > = 45 or < = 31) [12], [13] and respectively scored in pre-determined high and low ranges on the Albany Panic and Phobia Questionnaire, social phobia subscale (APPQSP) [14]. The high range on the APPQSP was 19 and above, with the bottom of this range being one standard deviation below the mean of an SAD population [14]. The low range was 16 and below, which represents the bottom 25% of a general population distribution. Use of the bFNES to select high and low socially anxious participants is well-established in social anxiety research [15]. The bFNES is a measure of fear of negative evaluation whereas the APPQSP measures fear of social situations. Including both measures as screening instruments made sure that participants feared social situations because of fear of negative evaluation and not for other reasons (e.g. because of fear of having a panic attack in public).

Participants also completed the Beck Depression Inventory (BDI) [16]. Individuals who scored above 20 on the BDI were excluded at the request of the local ethics committee. Participants were recruited using newspaper advertisements and through email within King's College London.

Table 1 shows the characteristics of high and low socially anxious participants. High and low socially anxious participants did not differ in age, gender, or ethnicity. As expected, high socially anxious participants scored higher than low socially anxious participants on measures of social anxiety, self-consciousness, self-focused attention, as well as depression.

**Table 1:** Characteristics of high and low socially anxious participants.

	High socially anxious (*n* = 48)	Low socially anxious (*n* = 48)	
	*M (SD)*/*N* (%)	*M (SD)*/*N* (%)	T/χ^2^
Age (years)	30.2 (11.9)	30.0 (8.9)	0.1
Female Sex	38 (79.2%)	32 (66.7%)	1.9
White Ethnicity	32 (66.7%)	24 (50.0%)	2.7
APPQ, Social Phobia Subscale	32.1 (13.8)	10.6 (8.6)	9.2***
Brief Fear of Negative Evaluation Scale	48.1 (6.2)	26.8 (6.6)	16.3***
Self-Consciousness Scale	62.8 (10.5)	41.5 (9.8)	10.3***
Self-Focused Attention Scale	22.2 (8.6)	8.8 (6.7)	8.6***
Beck Depression Inventory	9.7 (5.5)	3.9 (4.8)	5.6***

*Note.* *** *p*< .001; *M* =  Mean; *SD* =  Standard deviation; APPQ  =  Albany Panic and Phobia Questionnaire.

### Materials

#### Questionnaires

The *Brief Fear of Negative Evaluation Scale (bFNES)* and the *Albany Panic and Phobia Questionnaire, social phobia subscale (APPQSP)* were used to select the high and low social anxiety groups. The bFNES has 12 items. Scores range from 12–60 with higher scores indicating greater fear of negative evaluation. Internal consistency has been shown to be high and the scale demonstrated good test-retest reliability [13]. The APPQSP assesses fear in 10 social situations. Scores range from 0–80 with higher scores indicating greater social anxiety. The scale has good internal consistency and test-retest reliability [14].

Self-consciousness was measured using the private and public self-consciousness sub-scales of the *Self-Consciousness Scale (SCS)* [17]. Scores range from 0–28 for public self-consciousness and from 0–40 for private self-consciousness. Test-retest reliability for both sub-scales is good [17].

Self-focused attention was measured with the *Self-Focused Attention Scale (SFAS)* [18]. The SFAS is an 11-item measure assessing a tendency to focus on one's own arousal during social situations and self-focusing on one's own behavior. Items are summed to a total score ranging from 0–44. The scale has satisfactory internal reliability and convergent validity [18].

Levels of depression were measured using the *Beck Depression Inventory (BDI)* [16], [19]. It measures symptoms of depression on 21 items. The total score ranges from 0–63 with higher scores indicating greater depression. The BDI has good internal consistency and test-retest reliability [19].

Focus of attention during the experiment was measured on a 7-point bipolar scale ranging from -3 (*totally focused on myself/my body*) to +3 (*totally focused on my surroundings/the task*). Self-evaluation and anxiety during the experiment were measured with visual analogue scales ranging from 0 (*not at all*) to 100 (*totally*). For self-evaluation, participants rated how much they thought about how well or badly they were coming across.

#### Faces in a crowd task

This task aimed to assess the percept of being observed by other people by asking participants to estimate the proportion of people who were looking at them. On each trial participants were presented on a computer screen with 18 head and shoulders photographs of people. Some people were looking directly at the participant, while others were either turning their heads 45° to the left, 45° to the right, or down. The number of people directly looking at the participants ranged from 22% (4/18) to 78% (14/18). After each trial, participants were asked to estimate on a visual analogue scale (*Nobody (0%*) – *Everybody (100%))* the proportion of people who were looking at them.

Two picture sets were created (version a and b). Each picture set consisted of 18 people with a neutral facial expression, half of them female, half of them male. Fourteen people on the pictures were Caucasian. In each picture set, there was one Pacific Asian man and one Pacific Asian woman, one Indian/Pakistani/Bangladeshi woman, and one mixed (British-Black) man. In each picture the faces were arranged in a 5×4 matrix with the two central blocks being blank and the remaining 18 showing different people's faces. The two central blocks were left blank to make sure that participants would scan the periphery of the matrix and not just focus on the two central blocks. Four picture matrices were created for every number of people looking (four people looking: version 1, 2, 3, 4; five people looking: version 1, 2, 3, 4, etc., up to 14 people looking: version 1, 2, 3, 4) leading to a total number of 44 picture matrices for every picture set.

In each trial, a face matrix was presented for 2750 ms. Results of a pilot study (*N* = 7) indicated that this presentation time was long enough for people to be aware of the faces, but too short to be able to solve the task by counting the number of faces. Before each matrix appeared, a fixation cross was presented in the centre of the screen for 1000 ms. After each matrix was presented, a visual analogue scale appeared on the computer screen. Participants were instructed to move the indicator on the scale with their mouse so it recorded their estimate of the proportion of people who had been looking at them (*Nobody (0%)* – *Everybody (100%))*. After participants had made their estimate, a blank slide was presented for 3000 ms, before the next fixation cross, and then the next matrix came up. Each participant saw 44 face matrices. The order of presentation was random with the constraint that a matrix could only be shown once.

#### Mirror manipulation

Four mirrors were used (two mirrors with 70 cm width and 70 cm height and two mirrors with 70 cm width and 160 cm height). The two smaller mirrors were placed on the desk next to the monitor, the two bigger mirrors were placed on the floor next to the participants chair. The participants could see their reflection in the periphery of their eyes whilst doing the computer task.

### Procedure

All participants gave written consent and completed the APPQSP, BDI, SCS, and SFA. They were then given a practice block of 11 trials with the faces in a crowd task. Instructions were: *In the following computer task, you will be looking at several pictures with crowds of people, some of them are looking at you, some of them are not. Your task is to indicate the proportion of people who were looking at you*. Participants were asked to move the front legs of their chair to two defined spots on the floor to make sure that all the participants had the same distance to the screen. After the practice session, any remaining questions were answered. Participants were then asked to leave the laboratory and to complete a socio-demographic questionnaire. In the meantime, the experimenter set up the four mirrors with either the reflective side facing the participants (mirrors present condition) or the reflective side turned away from them (mirrors absent condition). On the way back to the laboratory, the experimenter gave participants the following instruction: *As there will be quite a lot of pictures to rate, you will have a short break during the computer task. During this break, I will ask you to complete a short questionnaire about how you were feeling during the task.* They then completed the first part of the faces in a crowd task, either in the mirrors present or mirrors absent condition. After the first part of the task, they were asked to leave the laboratory again and completed a scale assessing their focus of attention, self-evaluation, and anxiety during the first part of the task. The same sequence was repeated for the second part of the task, which was performed in the opposite mirror condition to that used in the first part. At the end, the experimenter explained the purpose of the study and reimbursed participants with £15.

## Results

### Validation of the faces in a crowd task

**Figure 1:**
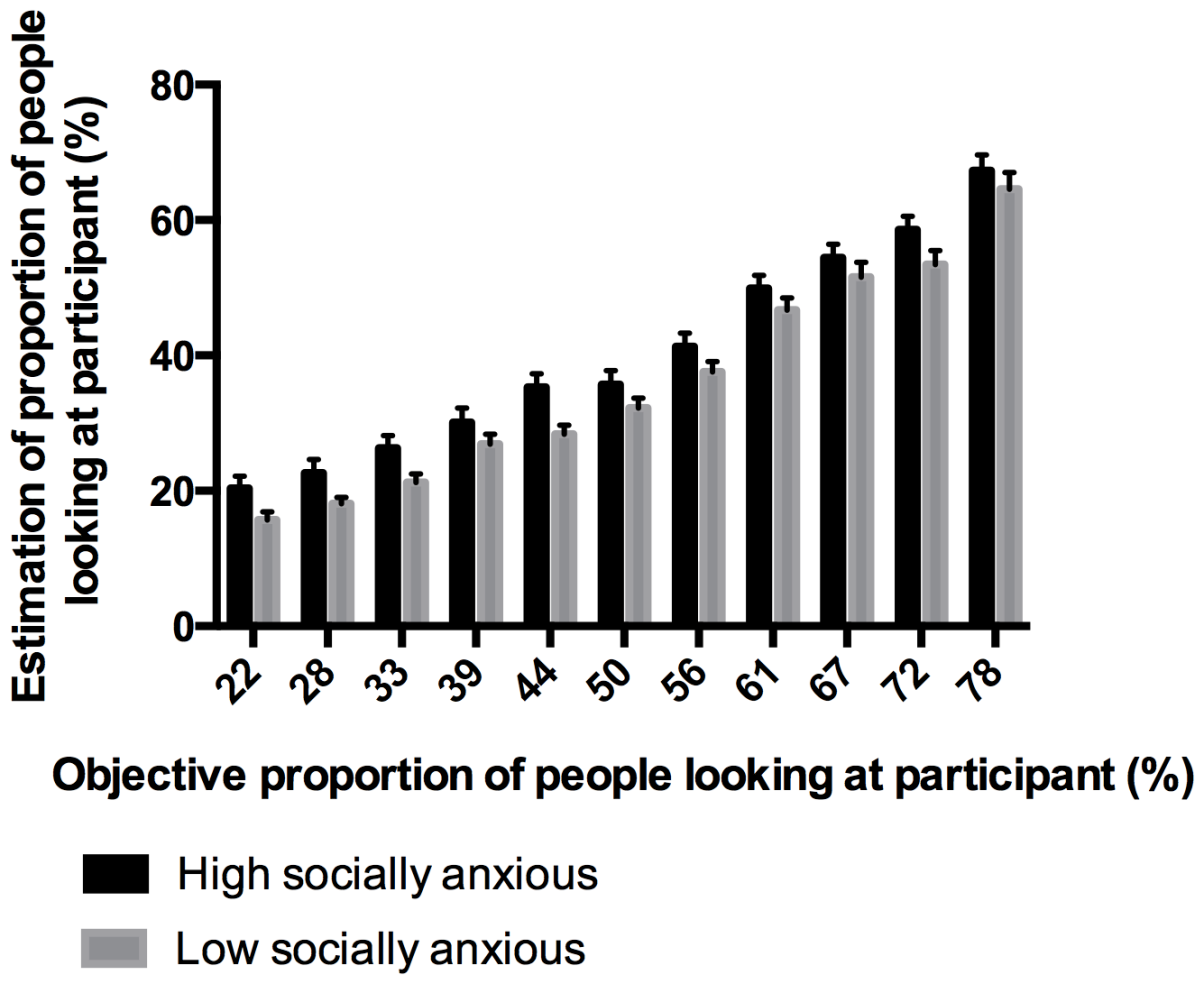
Increase of high and low socially anxious participants' estimates with increase of objective proportion of people looking in their direction. Error bars show standard errors.

In order to check that participants were processing the faces matrices, rather than simply guessing, we assessed whether participants' estimates of the number of people looking at them were correlated with the objective number. Mean scores for matrices depicting the same number of people looking at participants were computed. Figure 1 shows the relationship between the objective proportion of people looking towards the participants and participants' estimates of that proportion.

There was a positive correlation between estimates and the objective proportion of people looking towards the participant, in both high, *r* =  .987, and low socially anxious participants, *r* =  .985. It therefore appears that subjective ratings were at least partially based on processing the pictures. Overall, high and low socially anxious participants underestimated the proportion of people who were looking at them.

### Mirror manipulation check

It was expected that the mirror manipulation would increase self-focused attention. We were also interested to see whether it increased self-evaluation and anxiety. Two-way mixed ANOVAs with the between-subjects factor group (high/low socially anxious) and the within-subjects factor mirror (present/absent) were conducted to investigate the effects of the mirror manipulation on these variables. There were main effects of the mirrors for focus of attention, *F*(1, 94)  = 57.98, *p<* .001, η^2^ =  .38, and anxiety, *F*(1, 94) = 22.13, *p*< .001, η^2^ =  .19, indicating that participants were more self-focused and more anxious when the mirrors were present. There were also main effects of group for focus of attention, *F*(1, 94)  = 8.83, *p*< .01, η^2^ =  .09, and for anxiety, *F*(1, 94) = 38.41, *p*< .001, η^2^ =  .29, indicating that high socially anxious individuals were more self-focused and more anxious than low socially anxious individuals. The group × mirror interactions for focus of attention, *F*(1, 94) = 3.46, *p* =  .07, η^2^ =  .04, and anxiety, *F*(1, 94) = 2.71, *p* =  .10, η^2^ =  .03, did not reach significance, indicating that the self-focused attention and anxiety inducing effect of the mirrors did not differ significantly between the two groups.

For self-evaluation, the two-way ANOVA revealed a main effect of the mirrors, *F*(1, 94) = 15.09, *p<* .001, η^2^ =  .14, and a main effect of group, *F*(1, 94) = 25.79, *p*< .001, η^2^ =  .22, which were qualified by a group × mirror interaction, *F*(1, 94) = 8.12, *p*< .01, η^2^ =  .08. Separate paired *t*-tests within high and low socially anxious participants revealed that high socially anxious participants were significantly more self-evaluative when the mirrors were present, *t*(47)  = 4.11, *p*< .001. Low socially anxious participants did not significantly differ in self-evaluation in the two mirror conditions, *t*(47)  = 0.90, *p* =  .37.

Overall, the mirror manipulation enhanced self-focused attention and anxiety in high and low socially anxious individuals, but only enhanced self-evaluation in the high socially anxious participants. This finding is consistent with Clark & Wells' cognitive model [9], which proposes that self-focused attention and self-evaluation go hand in hand in individuals with high social anxiety, but not necessarily in individuals with low social anxiety. This is because high socially anxious individuals are said to have a particular tendency to use internal information (images, body sensations, etc.) to decide how they appear to others. In line with this model, in the mirrors present condition there was a significant correlation between self-focused attention and self-evaluation in high socially anxious individuals (*r* = −.362, *p* =  .001) indicating that the more self-focused they were, the more they reported engaging in self-evaluation. No such correlation was observed in low socially anxious individuals (*r* = −.057, *p* =  .702).

### Faces in a crowd task

A two-way ANOVA was conducted with the between-subjects factor group (high/low socially anxious) and the within-subjects factor mirror (present/absent) to investigate whether high and low socially anxious individuals differed in their estimates of the proportion of people looking at them and whether any difference was influenced by the mirror manipulation. Table 2 shows the means and standard deviations. In line with our hypothesis, a main effect of group, *F*(1, 94) = 5.85, *p* =  .02, η^2^ =  .06, indicated that high socially anxious individuals gave higher estimates for the proportion of people looking at them than low socially anxious individuals.

**Table 2:** High and low socially anxious participants' estimates of the proportion of people in the crowds who were looking at them.

	High socially anxious (*n* = 48)		Low socially anxious (*n* = 48)	
	Mirrors	No mirrors	Mirrors	No mirrors
	*M (SD)*	*M (SD)*	*M (SD)*	*M (SD)*
Estimation of proportion of people looking at participants (0–100%)	40.4 (12.2)	40.2 (11.2)	34.9 (9.3)	36.0 (8.1)

*Note. M* =  Mean; *SD*  =  Standard deviation.

Contrary to expectation, the interaction between group and mirror manipulation was not significant, *F*(1, 94) = 1.10, *p* =  .30, η^2^ =  .01, so there was no overall evidence that the magnitude of the difference in estimates between the groups was influenced by the mirror manipulation.

High socially anxious individuals scored higher on the BDI than low socially anxious individuals. To determine whether the group difference in estimates of being observed could be attributed to depression, rather than social anxiety, we performed a two-way (group × mirror) analysis of covariance with participants' BDI scores as the covariate. The main effect of group remained significant, *F*(1, 94) = 4.04, *p*< .05, η^2^ =  .04, suggesting that elevated levels of depression cannot explain why high socially anxious individuals estimated that more people were looking at them.

To check whether the objective number of faces in the displays influenced the magnitude of any social anxiety related effects, we also conducted a series of three-way ANOVAs with the third factor being the number of faces in the displays. There were no significant interactions involving social anxiety group and number of faces.

#### Post-hoc analysis

Several participants commented at the end of the experiment that they were very aware of the mirrors in the early part of the faces in a crowd task, but that after a while, they forgot that they were there. This raises the possibility that the effectiveness of the mirror manipulation faded as a session progressed. For this reason it was decided to conduct a post-hoc analysis in which “phase in the task” was included as a factor. A three-way mixed ANOVA with group (high/low socially anxious) as the between-subjects factor, and mirror (present/absent), and phase (trials 1 to 14, trials 15 to 30, trials 31 to 44) as within-subjects factors was conducted.

**Figure 2:**
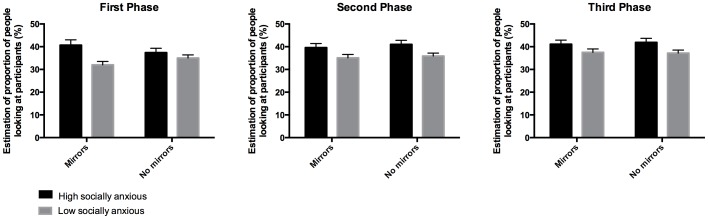
High and low socially anxious participants' estimates of the proportion of people in the crowds who were looking at them (0–100%) with and without mirrors present in the three phases of the experiment. Error bars show standard errors.

The main effect of group remained significant. In addition, there was also a main effect of phase, *F*(2, 188) = 9.19, *p*< .001, η^2^ =  .09, indicating that participants estimated that more people were looking at them as the task progressed. Importantly, there was also a significant phase × group × mirror interaction, *F*(2, 188) = 4.92, *p* =  .01, η^2^ =  .05. Figure 2 illustrates this interaction.

To further investigate this interaction, separate two-way (group, mirror) ANOVAs were conducted for each phase in the experiment. In the first phase, there was a main effect of group, *F*(1, 94) = 5.52, *p* =  .02, η^2^ =  .06, which was qualified by a group × mirror interaction, *F*(1, 94) = 7.84, *p*< .01, η^2^ =  .08. To further examine the group × mirror interaction in the first phase, separate independent *t*-tests were conducted for the mirrors present and absent conditions. When the mirrors were present, the two social anxiety groups significantly differed from each other, *t*(94) = 3.11, *p*< .01, with high socially anxious individuals estimating that more people were looking at them than low socially anxious individuals. When the mirrors were absent, there was no significant difference between the two groups, *t*(94) = 0.98, *p* =  .33. It therefore appears that in the first phase of the experiment, the group difference in individuals' estimates of the proportion of people who were looking at them was increased by the mirror manipulation. In the second and third phases of the experiment, there were main effects of group (second phase: *F*(1, 94) = 5.21, *p* =  .03, η^2^ =  .05; third phase: *F*(1, 94) = 4.15, *p* =  .04, η^2^ = .04), but no significant main effects of the mirror manipulation and no significant group × mirror interactions. The impact of the mirrors on estimates of the proportion of people looking at participants had therefore faded after phase one, with neither groups' estimates being influenced by the presence of the mirror.

### Rating times

The two-way and the three-way ANOVAs were repeated using rating times (ms) as the dependent variable. There were no significant main effects or interactions involving group or the mirror manipulation. There is therefore no evidence that either social anxiety or the mirror manipulation affected participants' engagement with the faces in a crowd task.

## Discussion

The present study showed that high socially anxious individuals estimate that a higher proportion of people in a crowd are looking at them than low socially anxious individuals do, even when the objective proportion of people who are looking at them is the same. Although it is still possible that high socially anxious individuals attract more attention in a crowd, it seems clear that part of their impression that “everyone is looking at me” is likely to arise from a difference in their perception. Our result is in line with previous studies that have used the single other person “cone of gaze” paradigm and shows that socially anxious individuals' enhanced perception of being observed by others extends to crowds, and not just to being observed by others out of the corners of their eyes.

We hypothesized that high socially anxious individuals' tendency to estimate that more people are looking at them may be a consequence of their well-established heightened levels of self-observation and evaluation. In particular, we suggested that they may be confusing self-observation and evaluation with scrutiny by others. From this theoretical position we deduced the prediction that the presence of mirrors would enhance the perception of “being looked at by everyone”. The overall pattern of results for the mirror manipulation did not support this prediction. However, there was some evidence that participants were less aware of the mirrors as the faces in a crowd task progressed. A post-hoc analysis was therefore conducted which showed that in the first phase of the experiment the mirrors had their predicted effect. As this analysis was post-hoc, the result needs to be confirmed in further studies, which would ideally use a stronger and more persistent manipulation.

Our results have several potential clinical implications. Patients with SAD may find it helpful to know that they may be estimating the extent to which they are the focus of others' attention as higher than people without SAD. Behavioral experiments can be planned in therapy to confirm this point. Tentatively, one might suggest that training in non-evaluative externally focused attention may further reduce the feeling of “being looked at by everyone”.

The present study has some limitations. First, it is an analogue study and we cannot be sure that the results will generalize to patients with SAD. A further study with a clinical sample is required to assess this. However, previous research has shown that in social anxiety, results from analogue studies frequently hold true in clinical samples [20], [21]. Second, both groups of participants underestimated how much they were being observed when compared to the objective proportion of people looking at them. We think this might have to do with the short presentation time for the task. Participants might not have had enough time to process the whole picture. They clearly estimated that more people were looking at them when objectively more people were looking at them (as shown in Figure 1), but they might not have been able to process all the people on the pictures. The fact that a practice effect was observed with both groups increasing their estimates as the experiment progressed is consistent with this explanation. An alternative explanation may be that low socially anxious individuals generally underestimate the extent to which they are the subject of other people's attention as this is not a major concern for them. There are certainly other examples where anxious individuals are more accurate than non-anxious individuals at detecting events when they are related to their fearful concerns. For example, panic disorder patients are often afraid that there may be something wrong with their hearts and they are more accurate at detecting their heart beats than individuals without such fears, who tend to underestimate the number of beats [22]. Moreover, prospective studies suggest that the higher, but more accurate, heart beat estimates of panic disorder patients play a role in maintaining the disorder [23]. Third, the potential role, if any, that enhanced estimates of being observed by others may play in the development of social anxiety problems has yet to be explored. Longitudinal studies would be required to determine whether it is a potential risk factor for the development of social anxiety or a cognitive characteristic that emerges later.
